# Successful Removal of an Ovarian Tumor by the Large Abdominal Section

**Published:** 1850-11

**Authors:** 


*Successful removal of an Ovarian Tumor by the large abdominal section.* By Dr. VAN BUREN, of New York.

In this case, which is stated to be the second successful operation of the kind which has occurred in this city, the patient was 21 years of age, unmarried, and the tumor of five years growth, and still increasing in size. It was purely a fibrous growth of the left ovary, and connected with the uterus by the broad ligament only, which was very much attenuated and elongated. The incision by which its removal was effected, was nearly thirteen inches in length, and the tumor weighed over seven pounds. The omentum was partially adherent to its surface, and on detaching its connections with the knife, three arteries (of the omentum) were divided, which required ligatures, both ends of which were cut off, the knots being returned into the abdomen. The pedicle of the tumor was surrounded by a solitary ligature, the ends of which were brought out of the external wound. This was closed by the "*Carlsbad insect pins,*" and the twisted suture, the favorite method of Dieffenbach, of Berlin, in cases where certain union by the first intention was desired.

The case progressed favorably ; not a solitary bad symptom occurred which was not readily controlled, and after a month's confinement to the bed, the patient was allowed to resume her ordinary occupations.

It should have been mentioned that the patient was suffering from an extreme degree of *procidentia uteri,* a consequence of the pressure exerted by the tumor from above. This was reduced before the operation, and retained in its place after the patient's recovery by a globular caoutchouc pessary, about two and a half inches in diameter, which answered its purpose admirably.

Dr. Van Buren attributes the successful result of his case, 1st, To its exceedingly promising character from the great mobility and slender attachments of the tumor; 2d, To the employment of chloroform during the operation, by which all shock was prevented; and 3d, By the free use of opium to obviate peritonitis.—*N. Y. Jour. of Med., March,* 1850.

